# Case report of hyperglycemic nonketotic chorea with rapid radiological resolution

**DOI:** 10.1186/s12880-017-0228-2

**Published:** 2017-09-12

**Authors:** H.M.M.T.B. Herath, S.P. Pahalagamage, Sunethra Senanayake

**Affiliations:** National hospital, Colombo, Sri Lanka

**Keywords:** Hyperglycemic nonketotic chorea, Rapid radiological resolution

## Abstract

**Background:**

Hemichorea is a rare manifestation of nonketotic hyperglycemia that usually affects elderly Asian women with poor glycemic control. Non-contrast computerized Tomography and T1- weighted Magnetic Resonance Imaging shows characteristic hyperintense basal ganglia lesions.

**Case presentation:**

A Fifty-seven year old Sri Lankan female presented with a two-day history of right upper limb chorea. She had been diagnosed with diabetes mellitus one year ago, but was not on any treatment and did not have any micro vascular or macro vascular complications. Random blood sugar was 420 mg/dl and full blood count, liver function tests, renal function tests, inflammatory markers, thyroid function tests, Urine protein / creatinine ratio, electrocardiogram and 2D Echo were normal. Arterial blood gas did not show acidosis and ketone bodies were not detected in urine. Non-contrast computerized Tomography brain on day 1 showed left side hyperdense lentiform and caudate nuclei and MRI on day 3 showed slightly high signal intensity of left side basal ganglia on T1- weighted images and low signal intensity on T2-weighted and Fluid-attenuated inversion recovery images. She was started on insulin and a low dose of clonazepam and glycemic control was achieved on day 3. Two days later, the chorea completely disappeared. CT brain was repeated 4 days and 10 days following glycemic control, which showed rapid resolution of CT changes. Clonazepam was stopped in 2 weeks and chorea did not recur.

**Conclusion:**

This is a rare manifestation of diabetes in Sri lanka and diagnosing this rare entity will direct clinicians to achieve optimum glycemic control as the treatment which will lead to rapid clinical response without any other medications. In this case report we high light that with the clinical improvement, repeating a CT scan even after a very short period like 2 weeks will show rapid radiological resolution. This repeat imaging can also be useful to confirm the diagnosis, which will minimize unnecessary investigations and treatments. Further cases of hyperglycemic nonketotic chorea with brain imaging performed within short intervals is needed to evaluate the nature of rapid radiological changes, which will be useful to understand the pathology of this condition.

## Background

Hemichorea is a rare manifestation of nonketotic hyperglycemia that usually affects elderly Asian women with poor glycemic control. The pathophysiology of hemichorea in hyperglycemia is controversial but these patients display distinct neuroimaging features. Non Contrast Computerized Tomography (NCCT) and T1- weighted Magnetic resonance imaging (MRI) show characteristic hyperintense basal ganglia lesions but the T2-weighted brain MRI findings vary [1–3]. In this case we followed up a patient with nonketotic hyperglycemic chorea with serial CT scans performed in very short intervals which revealed rapid radiological resolution.

## Case presentation

**Fig. 1:**
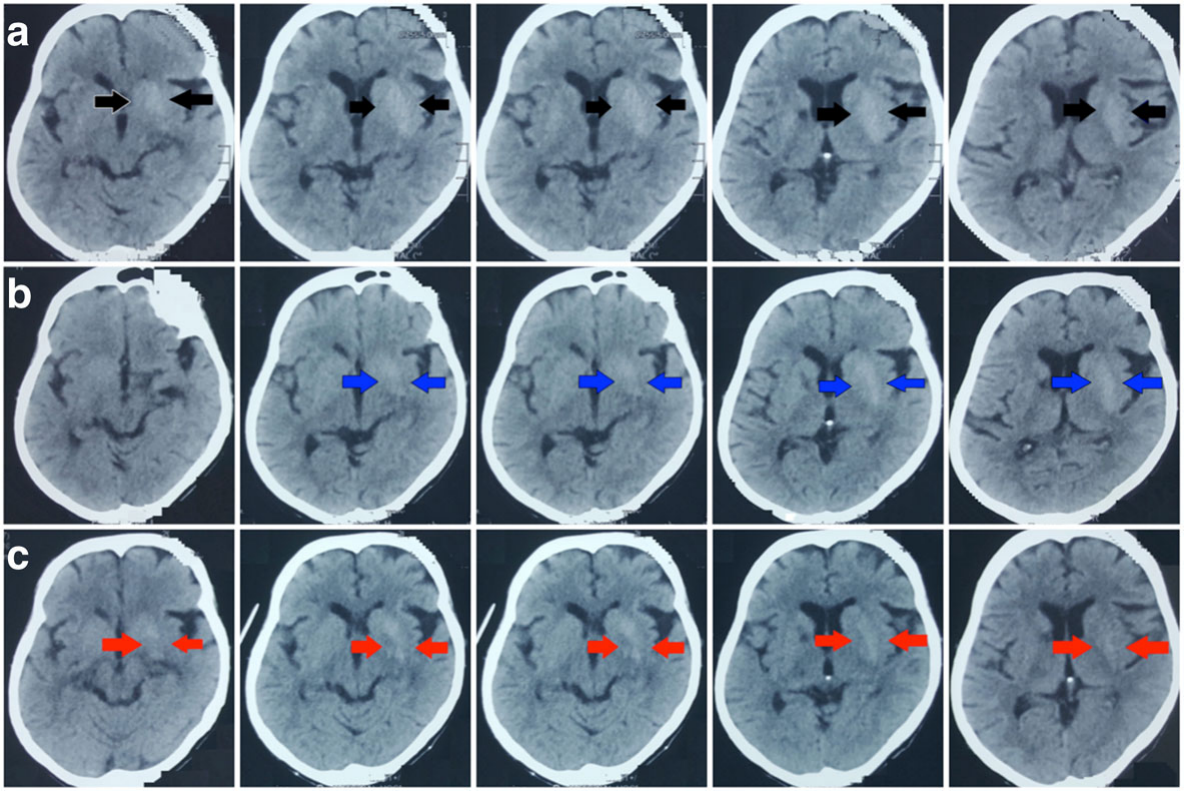
Serial NCCT brain showing resolution of left side high intensity basal ganglia. **a**: Day 1 NCCT brain showing left side hyperdense lentiform and caudate nuclei shown by black arrows. **b**: NCCT brain 4 days after glycemic control showing rapid resolution of left side hyperdense lentiform and caudate nuclei (The hyperdense area is shown by blue arrows). **c**: NCCT brain 10 days after glycemic control showing rapid resolution of left side hyperdense lentiform and caudate nuclei (The initial hyperdense area is shown by red arrows, which has resolved by day 10)

**Fig. 2:**
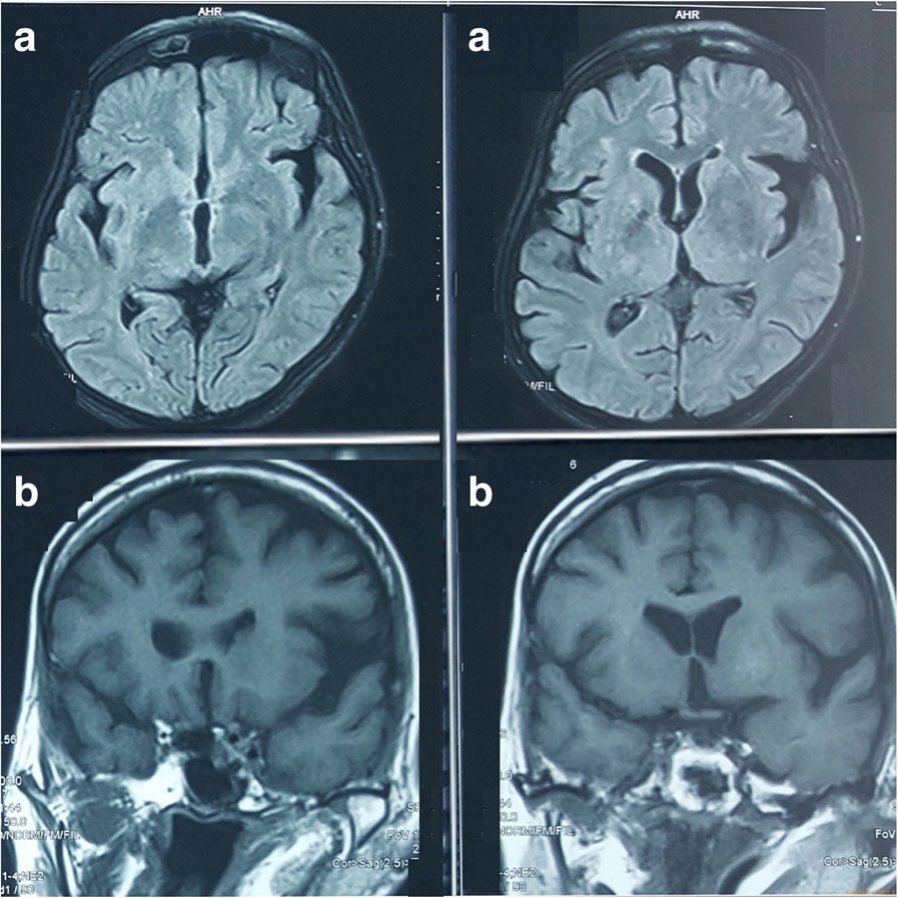
**a**: MRI on day 3 showing slightly low signal intensity of left side basal ganglia on T2- weighted images. **b**: MRI on day 3 showing slightly high signal intensity of left side basal ganglia on T1- weighted images

A Fifty-seven year old Sri Lankan female presented with a two-day history of acute onset right upper limb chorea. She had been diagnosed with diabetes mellitus one year ago, but was not on any treatment and did not have any micro vascular or macro vascular complications. On examination, right upper limb chorea was evident and the rest of the nervous system examination was normal including fundoscopy. Random blood sugar on admission was 420 mg/dl. Full blood count, liver function tests, renal function tests, inflammatory markers, thyroid function tests, Anti nuclear antibody, Urine protein / creatinine ratio, electrocardiogram and 2D Echo were normal. Arterial blood gas did not show acidosis and ketone bodies were not detected in urine. NCCT brain on day 1 showed left side hyperdense lentiform and caudate nuclei(Fig. 1). MRI on day 3 showed slightly high signal intensity of left side basal ganglia on T1- weighted images and low signal intensity on T2-weighted and Fluid-attenuated inversion recovery images(Fig. 2). Diffusion weighted imaging and Susceptibility weighted imaging were normal. She was started on insulin and a low dose of clonazepam (0.5 mg nocte) and glycemic control was achieved on day 3. Two days later, the chorea completely disappeared. NCCT was repeated 4 days and 10 days following glycemic control, which showed rapid resolution of CT changes (Fig. 1). Clonazepam was stopped in 2 weeks and chorea did not recur.

## Discussion

In this case, the patient had hemichorea, nonketotic hyperglycemia and hyperintense caudate and lentiform nuclei on NCCT and T1-MRI supporting the diagnosis of hyperglycemic nonketotic chorea. Several hypotheses have been put forward to explain the mechanism of chorea including relative dopaminergic hypersensitivity, vascular insufficiency causing transient ischemia to basal ganglia and shifting of cerebral metabolism to the anaerobic pathway causing reduction in both GABA and acetylcholine with metabolic acidosis leading to basal ganglia dysfunction [1, 2, 4]. As in our case, in most of the cases the chorea ameliorated completely with good glycemic control [1, 5] suggesting a reversible pathology.

Lai, P.H. at el., evaluated neuroimaging of 10 patients with nonketotic hyperglycemic hemichorea in primary diabetes mellitus and CT and T1-weighted MR images showed lesions of the putamen and/or caudate unilaterally or bilaterally. Lin, J.J. and colleagues report 7 similar patients with CT showing an increased density in the contralateral putamen and/or caudate and MRI revealing abnormal hyperintensity on T1-weighted and hypointensity on T2-weighted images [5]. The theories for characteristic radiological findings are abnormal deposition of calcium in neurons or glial cells, petechial hemorrhages from small vessels, extravascular hemosiderin deposition and gemistocyte (swollen reactive astrocytes having rich protein content) accumulation following acute injury [4]. Lin, J.J. and colleagues followed up 7 patients and the lesions on CT and MRI showed complete resolution within 3 months and 11 months, respectively [5]. In one literature survey, 19 out of 22 patients who had a follow-up brain MRI after an interval of 2 months – 1.5 years, the high signal intensity basal ganglia lesions resolved along with the improvement in chorea [1]. In another case series, 8 out of 9 patients underwent follow up CT scans within an interval of 1–18 months and the hyperdense striatal lesions had disappeared completely or near-completely [6].

In our patient we did serial CT scans over two weeks, which showed rapid resolution. As mentioned above, we came across on very few case reports in literature where the progression of radiological manifestations was monitored, but in this case we did CT scans in very short intervals. Previous case reports describe rapid clinical improvement but in this case with the resolution of chorea following glycemic control we also noticed the improvement of the CT scan within a very short period of few days. The rapid resolution of radiological changes suggests that the pathological changes in the brain parenchyma in hyperglycemic nonketotic chorea are rapidly reversing.

## Conclusion

The patient had poorly controlled diabetes without any complications, suggesting that chorea might not be related to microvascular or macrovascular complications of diabetes. This is a rare manifestation of diabetes in Sri lanka and diagnosing this rare entity will direct clinicians to achieve optimum glycemic control as the treatment which will lead to rapid clinical response without any other medications. In this case report we high light that with the clinical improvement, repeating a CT scan even after a very short period like 2 weeks will show rapid radiological resolution. This repeat imaging can also be useful to confirm the diagnosis, which will minimize unnecessary investigations and treatments. Further case reports and case series of hyperglycemic nonketotic chorea with brain imaging performed within short intervals is needed to evaluate the nature of rapid radiological changes which will be useful to understand the pathology of this condition.

## Abbreviations

MRI: Magnetic Resonance Imaging
NCCT: Non-Contrast Computerized Tomography
